# Repeated positron emission tomography tracing neutrophil elastase in a porcine intensive-care sepsis model

**DOI:** 10.1186/s40635-025-00721-3

**Published:** 2025-02-04

**Authors:** Frida Wilske, Olof Eriksson, Rose-Marie Amini, Sergio Estrada, Helena Janols, Amina Khalil, Anders Larsson, Miklós Lipcsey, Sara Mangsbo, Jonathan Sigfridsson, Jan Sjölin, Paul Skorup, Anders Wall, Viola Wilson, Markus Castegren, Gunnar Antoni

**Affiliations:** 1 Department of Medical Sciences, Infectious Diseases, Uppsala University, Uppsala, Sweden; 2 Science for Life Laboratory, Department of Medicinal Chemistry, Uppsala University, Uppsala, Sweden; 3 Department of Immunology, Genetics and Pathology, Uppsala University, Uppsala, Sweden; 4 Department of Medicinal Chemistry, Uppsala University, Uppsala, Sweden; 5 Department of Medical Sciences, Clinical Chemistry, Uppsala University, Uppsala, Sweden; 6 Department of Surgical Sciences, Anaesthesiology and Intensive Care, Uppsala University, Uppsala, Sweden; 7 Hedenstierna Laboratory, Department of Surgical Sciences, Uppsala University, Uppsala, Sweden; 8 Department of Pharmacy, Uppsala University, Uppsala, Sweden; 9 Department of Surgical Sciences, Molecular Imaging and Medical Physics, Uppsala University, Uppsala, Sweden; 10 CLINTEC, Karolinska Institutet, Stockholm, Sweden; 11 Centre for Clinical Research Sörmland, Uppsala University, Uppsala, Sweden

**Keywords:** Porcine, Sepsis, PET, Neutrophil elastase, *E coli*, [^11^C]NES, [^11^C]GW457427

## Abstract

**Background:**

Neutrophil granulocytes are important parts of the defence against bacterial infections. Their action is a two-edged sword, the mediators killing the intruding bacteria are at the same time causing tissue damage. Neutrophil activation is part of the dysregulated immune response to infection defining sepsis and neutrophil elastase is one of the powerful proteases causing both effects and damage. Inhibition of neutrophil elastase has been tried in sepsis and ARDS, so far with inconclusive results.

**Methods:**

We used positron emission tomography (PET) combined with computed tomography (CT) and the selective and specific neutrophil elastase inhibitor PET-tracer [^11^C]GW457427 ([^11^C]NES), in an intensive care unit porcine *Escherichia coli* sepsis model with the primary aim to visualise the biodistribution of neutrophil elastase in the initial acute phase of the septic reaction. Repeated PET–CT investigations were performed before and after induction of sepsis.

**Results:**

At baseline [^11^C]NES uptake was found in the bone marrow, spleen and liver. The uptake in the bone marrow was markedly increased two hours into the sepsis, whereas in spleen and liver the uptake was not as markedly changed compared to baseline. At 4 h after the sepsis induction [^11^C]NES in the bone marrow decreased while the uptake increased in the spleen, liver and lungs.

**Conclusion:**

The neutrophil elastase PET-tracer [^11^C]NES is a novel and unique instrument to study the acute innate neutrophil immune response in sepsis and associated vital organ failure. We here present images and quantitative data of the neutrophil elastase distribution the first hours of acute experimental sepsis. Surprisingly, a pronounced increase of neutrophil elastase was found in the bone marrow 2 h into the sepsis reaction followed at 4 h by increase in the liver, spleen and lungs and a concomitant reduction of the tracer uptake in bone marrow.

**Supplementary Information:**

The online version contains supplementary material available at 10.1186/s40635-025-00721-3.

## Background

Sepsis is defined as a life-threatening organ dysfunction caused by a dysregulated host response to infection [1]. It is estimated that sepsis is responsible for 11 million deaths per year globally [2]. Significant research effort has been devoted to understanding the dysregulated immune response and develop immunomodulatory treatments, but so far, no such treatment options have improved outcome in multicentre randomised trials. This is probably due to the heterogeneity of the septic syndrome but also to the knowledge gaps still existing regarding the interactions between the highly complex inflammatory reaction and the development of vital organ failure in sepsis [3].

Neutrophils are important cells in the innate immune response as they are the most abundant white blood cells and react quickly, initiating a fierce response igniting the clinical sepsis manifestations. Neutrophil elastase (NE) is only produced by neutrophils [4]. Myeloperoxidase (MPO) is found in the azurophilic granule alongside with NE, but is also produced by monocytes, macrophages, and other body cells [5]. The main source of mature neutrophils is the bone marrow. These neutrophils can be released quickly and increase the circulating amounts of neutrophils in the blood by a tenfold within hours [6].

Although neutrophils are important in the defence against bacteria, they also cause many of the sepsis-related clinical symptoms. NE has, if uncontrolled, potentially devastating effects on tissues [7, 8]. Neutrophil migration and trapping in the capillaries of the lungs is considered an important mechanism leading to organ failure, i.e. acute respiratory distress syndrome (ARDS) and disseminated intravascular coagulation (DIC) [9–11]. One main effect is due to endothelial injury caused by NE increasing the vascular permeability and activation of the coagulation cascade leading to micro-thrombosis and reduced blood flow in critical organs [12].

Elastase inhibitors have been investigated as treatment in sepsis to reduce the inflammatory response of the immune system. The selective NE inhibitor sivelestat has shown some efficacy in ARDS and DIC after systemic administration, yielding shorter intensive care unit (ICU) stay compared with non-treated patients [13]. Promising results with sivelestat have also been obtained in preclinical studies of sepsis [14, 15]. All together this shows that neutrophils are important in sepsis, with a dual role of simultaneously removing pathogens and contributing to tissue damage. This complicates the use of pharmacological interventions directed towards ameliorating neutrophil activity. The knowledge of the intricate interplay between cytokines, chemokines and neutrophil activity in the early phase of sepsis is incomplete.

We hypothesised that it would be possible to study the biodistribution of neutrophils by tracing a neutrophil-specific protein in repeated positron emission tomography combined with computed tomography (PET–CT) during the first hours in sepsis. In this study we used PET–CT and the selective and specific NE PET-tracer [^11^C]GW457427, coined [^11^C]NES, to dynamically study the neutrophil mediated inflammatory response in a large animal intensive care bacteraemic sepsis model [16–18]. To our knowledge, this is the first time the early neutrophil response to sepsis has been visualised with PET.

## Methods

### Aim, design and setting

**Fig. 1:**
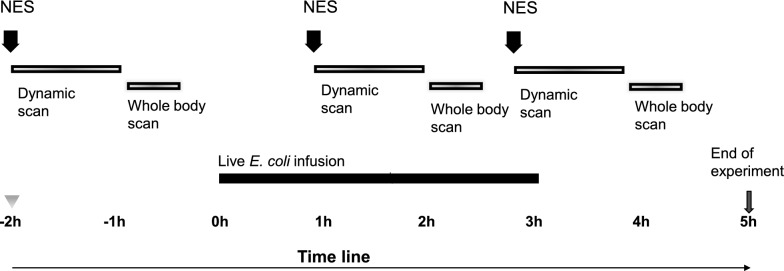
A timeline of the experimental setting. All animals were anaesthetised, prepared and transported to the PET-camera. At -2 h the first scan was started with an injection of [^11^C]NES and a 60 min dynamic scan followed by a 20–24 min whole-body scan. At 0 h sepsis was induced by the infusion of live *E. coli* during three hours. At 1 h the second scan was started with an injection of [^11^C]NES followed by the same dynamic and static protocol. For three of the animals a third scan was performed starting at 3 h. The experiment was terminated at 5 h

The aim of this study was to describe the distribution of neutrophil granulocytes by tracing NE in a porcine *Escherichia coli* (*E. coli*) model of sepsis using PET–CT and the selective NE PET-tracer [^11^C]NES [16, 19]. The experiment took place at the Hedenstierna laboratory, a facility for large animal intensive care research and the PET-centre at Uppsala University hospital. A timeline for the experiment is shown in Fig. 1.

### The subjects and intensive care protocol

Seven healthy land-breed pigs of both sexes weighing between 25 and 30 kg were used (P1–P7). They arrived at the facility in the morning and were immediately anaesthetised, tracheotomised and mechanically ventilated. The animals were handled and prepared as previously reported [19] and described in detail in Supplement 1.

### Interventions

Heart rate, mean arterial pressure (MAP) and mean pulmonary arterial pressure (MPAP) were monitored continuously. Cardiac output (CO) was measured hourly through thermodilution, and airway pressures including wedge pressure were registered hourly. Cardiac index was calculated as CO/body surface area (BSA) and pulmonary vascular resistance index (PVRI) was calculated as (MPAP-wedge pressure) * 80/CI. BSA was calculated using Meeh’s formula: BSA = k * body weight (kg) ^(2/3)^ [20].

Arterial blood gases were drawn hourly and analysed for oxygen pressure, carbon dioxide pressure, lactate, glucose and haemoglobin. Intensive care interventions were made according to Supplement 2. The depth of sleep, signs of pain and shivering were continuously observed by trained personnel and when needed, the animals were given additional doses of morphine, ketamine or rocuronium bromide.

### Ethical statement

The pigs were given food and drink ad libitum until one hour before arrival to the research facility where they immediately were anaesthetised. They were kept under deep anaesthesia until killed at the end of the experiment. Every effort was made to reduce suffering and the experiment was approved by the Animal Ethics Board in Uppsala, Sweden, permit number 5.8.18–08592/2019, supplementary decision made 2022–04-25. The study was designed with consideration of Minimum Quality Threshold in Pre-clinical Sepsis Studies and reported in coherence with the ARRIVE 2.0 guidelines [21, 22].

### Blood sample analysis

The arterial blood gases and the venous blood or plasma samples were analysed for oxygen pressure, carbon dioxide pressure, lactate, glucose, haemoglobin blood cell count, creatinine, tumour necrosis factor- alfa (TNF-α) and NE. The analysis methods are described in detail in Supplement 3.

### Bacteria

A clinical isolate of *E. coli* was prepared as described in Supplement 4. The bacteria were given as an intravenous (i.v.) infusion of 8.3 log_10_ colony forming units (CFU) × hour (h)^−1^ for three hours. The infusion was changed every hour to assure that the bacteria remained in log-phase.

### Radiochemistry

[^11^C]NES ([^11^C]GW457427) was produced as earlier described and obtained with a radiochemical purity higher than 95% [16]. Usually around 2 gigabecquerel (GBq) of activity was obtained at the end of synthesis. The positron emitting radionuclide carbon-11 has a short half-life of only 20.4 min, which allows repeated administrations of [^11^C]NES in the same animal on the same day, as each tracer administration will have decayed by more than 98% after 2 h (6 radioactive half-lives). Up to three synthesises were made per experimental day around 2 h apart, to enable a baseline scan and one or two follow-up scans after induction of sepsis. [^11^C]NES passes through the neutrophil cell membrane, and thus binds to both extracellular and intracellular NE.

### In vivo [^11^C]NES PET–CT examinations in pigs

For an overview of the PET–CT protocol, see Fig. 1. The pig was positioned in a supine position on the PET–CT scan bed. Next, an i.v. injection of [^11^C]NES at a dose of 12 megabecquerel (MBq) × kg^−1^ (around 300–360 MBq) was administered, followed by a 60-min dynamic PET-scan also functioning as a stabilisation period for tracer uptake and distribution. A whole-body CT scan was performed for attenuation correction and anatomical co-registration. Thereafter, a whole-body PET-scan lasting 20–24 min was performed. For more details see Supplement 5.

After the first PET-scan was finalised, experimental sepsis was induced by an intravenous bacterial infusion as described above. One hour after the start of the bacterial infusion, a second injection of [^11^C]NES was administered. At this time point, around three hours had passed from the first [^11^C]NES injection, and negligible radioactivity (< 0.3%) remained in the body. After 60 min of dynamic PET-scanning and stabilisation the whole-body PET and CT scans were repeated as described above.

Finally, in three of the pigs a third injection of [^11^C]NES was administered around 2 h after the second injection. As before, the [^11^C]NES injection was followed by dynamic PET-scan for stabilisation and static whole-body PET–CT scans.

### Image analysis

PET–CT image analysis was performed using the PMOD Base Functionality (PBAS) tool (PMOD Technologies LLC, Zurich, Switzerland). Manual segmentation and delineation of the lungs, bone marrow, liver and spleen were performed with assistance by CT images. Tracer uptake was quantified in Becquerels per cubic centimetre (Bq × (cc)^−1^) and subsequently converted to standardised uptake values (SUV) by correcting for the total administered dose (in kBq) and body weight (in kg). The unit is thus g/ml, but when the density as here can be approximated to 1 g/ml SUV becomes a unitless measure of PET-tracer uptake in tissues which can be compared within and between individuals.

### Calculations of inflammatory volume of the lung

The PET–CT images of the lungs were further analysed in more detail. The volume of the lungs with inflammation was calculated from CT-images using Hounsfield units (HU) and from PET based on SUV. Segmentations of the lungs were applied on the CT-image; a range of HU between − 980 and − 150 to − 50, to cover the entire lung including parts affected by atelectasis. The upper limit (− 150 to − 50) was adjusted to make the delineation as similar as possible before and after induction of sepsis. Within the segmented lung tissue a cutoff was set to include SUV above1.3 or 1.6, whereupon a region was delineated and subsequently used to exclude spillover of uptake from bone marrow in the ribs and other tissues close to the lung. The total inflammatory volume (TIL) was calculated by multiplying SUV_mean_ with the lung volume having a SUV above the selected cutoff. The selection of the SUV cutoff was set based on the background uptake in the baseline scan before sepsis was induced and was then fixed for the scans at 2 h and 4 h to exclude the tissue with atelectasis and gravitational effects from the TIL. Image processing and analysis was performed with the software Affinity 3.0.5 (Hermes medical solutions, Stockholm, Sweden).

### *Blocking of extracellular NE *in vivo

One of the animals, P6, was administered the extracellular NE inhibitor sivelestat (400 mg; 14 mg × kg^−1^) as an i.v. infusion (400 mg in 50 mL DMSO, 2 mL × min^−1^, 10 min in total) 30 min prior to the third [^11^C]NES injection.

### Bone marrow sampling

From subjects P6 and P7, a bone marrow biopsy was taken from the femoral bone before (− 2h) and after (3h) sepsis induction. The object glasses with imprints and the biopsies were stained both with standard stain for cell count and with antibodies targeting MPO and NE, respectively. See Supplement 6.

### *Blocking of NE in purified human neutrophils *in vitro

Neutrophils were purified and either lysed or intact incubated with [^11^C]NES in the presence of three different elastase inhibitors, GW457427 (NES), GW311616 and sivelestat. The trapped radioactivity was measured in an in-house built scintillation counter of NaI type and corrected for radioactive decay. For details see Supplement 7.

### Statistics

Statistica 14.0 (StatSoft, Tulsa, OK, USA) was used for most statistic calculations. Mean and standard deviations (SD) were used (unless otherwise stated) and a *p*-value < 0.05 was considered significant. Because of the biological variations we chose the non-parametric Wilcoxon test when testing for differences before and after sepsis induction. For the analysis of blocking of NE in vitro GraphPad Prism 10.4.0 was used for a *t*-test and Fig. 8.

## Results

Seven pigs were included in the experiment, with a weight of 28.9 ± 0.9 kg. One animal died soon after the start of bacteraemia and was excluded from further analyses. The animals were given a mean total dose of 8.6 ± 0.2 log^10^ CFU *E.coli* i.v. to induce sepsis. Table 1 shows the physiological and biochemical reaction to the septic insult, leading to significant changes in vital organs. All animals developed a rapid septic reaction, characterised by increased core temperature and heart rate, and concurrently reduced arterial blood oxygenation and dynamic pulmonary compliance (Table 1). The acute hypodynamic septic shock phase was evident in the need for high-dose vasopressor support, i.e. noradrenaline, disguising the decrease in MAP. The pulmonary circulation was acutely affected with increasing MPAP and PVRI in conjunction with decreased CI leading to a markedly reduced pulmonary blood flow. The inflammatory biomarker TNF-α was markedly increased, peaking one hour after the induction of sepsis. Of the six surviving animals, four required administration of noradrenaline due to shock.

**Table 1:** Inflammatory, respiratory and circulatory measurements developing during sepsis

		− **2 h**	**1 h**	**5 h**	***p***
Temperature	°C	38.9 ± 0.7	39.6 ± 0.7	40.2 ± 0.7	a: < 0.05 b: < 0.05
Plasma TNF-α	ng × mL^−1^	108.5 ± 40.0	2371.2 ± 1102.8	523.8 ± 185.0	a < 0.05 b < 0.05
Plasma elastase	ng × mL^−1^	6.84 ± 4.07	4.76 ± 0.76	5.22 ± 1.23	a = 0.25 b = 0.60
CI	L × min^−1^ × m^−2^	2.80 ± 1.27	2.03 ± 0.78	3.13 ± 0.50	a < 0.05 b = 0.46
MAP	mmHg	78 ± 12	86 ± 10	93 ± 12	a = 0.40 b < 0.05
MPAP	mmHg	21 ± 4	43 ± 5	30 ± 5	a < 0.05 b < 0.05
PVRI	dynes × s^−1^ × cm^−5^ × m^−2^	234 ± 137	807 ± 284	546 ± 284	a < 0.05 b < 0.05
Arterial lactate	mmol × L^−1^	1.6 ± 0.4	1.7 ± 0.5	0.9 ± 0.1	a = 0.83 b < 0.05
Noradrenaline	μg × kg^−1^ × min ^−1^	0.03 ± 0.05	0.27 ± 0.22	0.12 ± 0.13	a = 0.07 b = 0.35
PaO_2_/FiO_2_	mmHg	396 ± 49	311 ± 66	189 ± 81	a < 0.05 b < 0.05
Compliance	mL × cmH_2_O^−1^	23.9 ± 3.2	19.1 ± 2.3	19.1 ± 2.8	a < 0.05 b < 0.05

The sepsis reaction summarised with baseline (− 2 h), peak (1 h) and end (5 h) values of chosen inflammatory, respiratory and circulatory measurements. The p-values are results from Wilcoxon’s signed-ranked test comparing paired values between, a: − 2 h and 1 h and b: − 2 h and 5 h

All shown as mean ± standard deviation

TNF, Tumour necrosis factor; CI, Cardiac index; MAP, Mean arterial pressure; MPAP, Mean pulmonary arterial pressure; PVRI, Pulmonary vascular resistance index; PaO_2_/FiO_2_, Partial pressure of oxygen in arterial blood; Compliance, Static airway compliance

### Neutrophils in blood

**Fig. 2:**
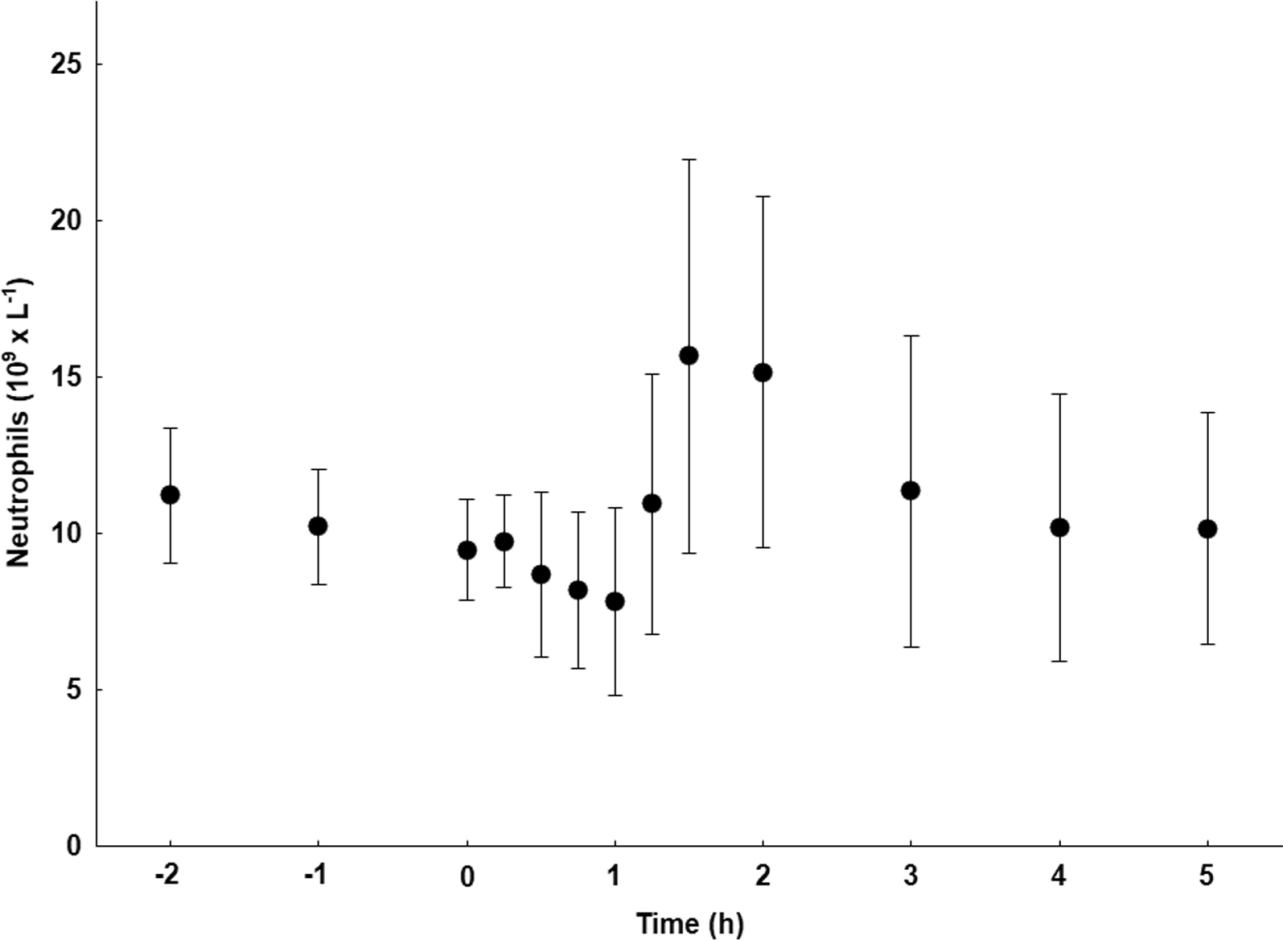
The amount of neutrophils in peripheral blood over time. Mean ± standard error (SE)

The neutrophil counts in blood showed, although with a large individual variation a rapid decrease from baseline after sepsis induction with the lowest level at 1 h, followed by a steady increase during 30 min and then a slow decline to nearly baseline level (Fig. 2). NE in plasma decreased by approximately 30% one hour in to sepsis as seen in Table 1.

### PET-results

At baseline NE was found mainly in lymphoid tissues such as the bone marrow and to a lesser extent in the spleen which is the normal distribution of [^11^C]NES based on previous pig scan and thus expected. This binding is consistent with the presence of neutrophil reservoirs in these tissues. Radioactivity was also present in the intestinal lumen at baseline (before disease induction) demonstrating biliary excretion of labelled metabolites via the liver.

**Fig. 3:**
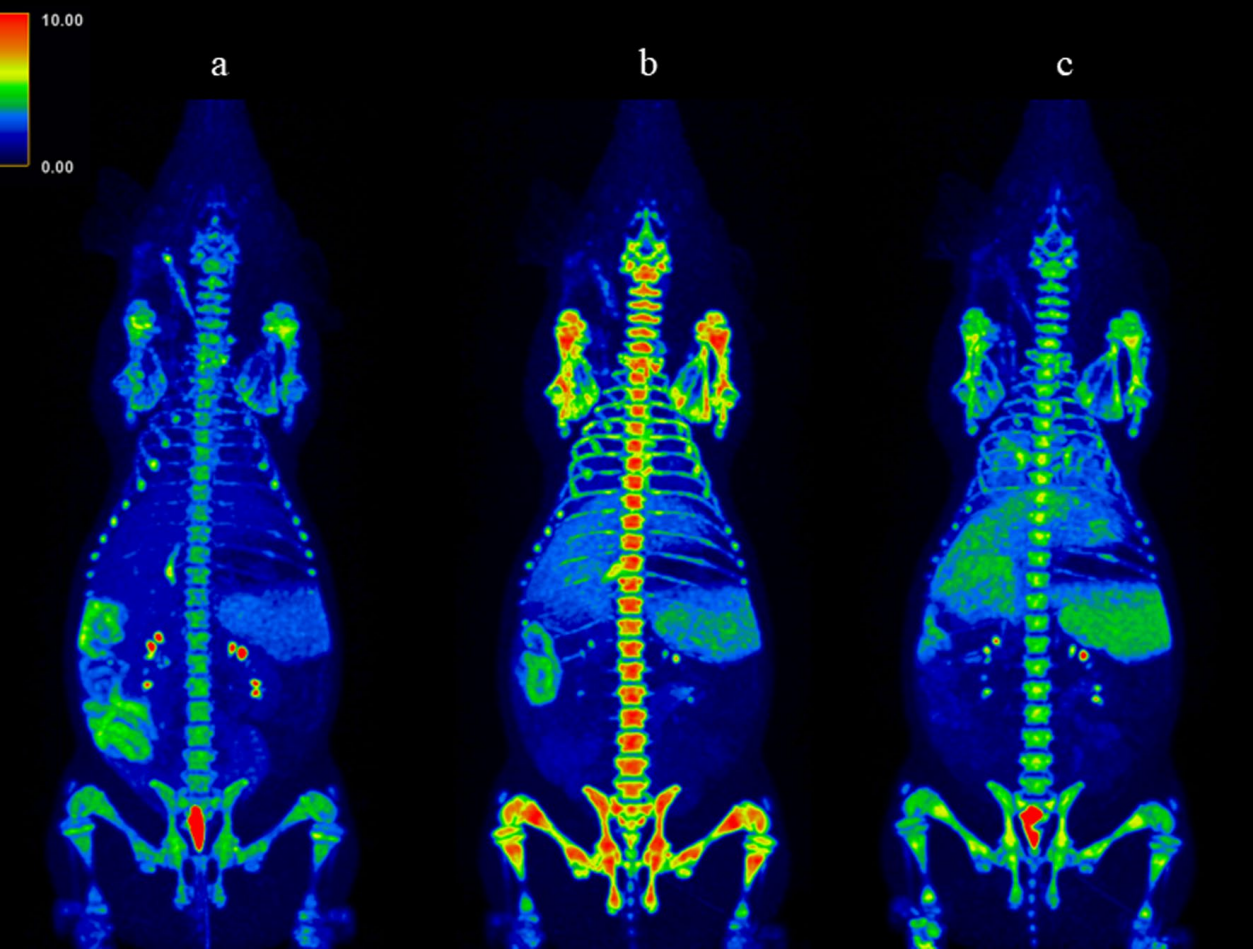
Coronal maximum intensity projection (MIP) PET–CT scans with [^11^C]NES. **a** − 1 h, **b** 2 h, **c**) 4 h. All images are corrected to SUV = 10 and thus directly comparable. The tracer uptake in the bone marrow is significantly increased from baseline − 2 h to 2 h and then decreases. The uptake in the liver is increasing during the progress of sepsis. In the spleen the uptake is increasing at 4 h. We see the elimination of tracer from the kidneys to the urinary bladder and from the biliary tract to the intestine, clearly visible at -2 h, but less distinct after sepsis induction

**Fig. 4:**
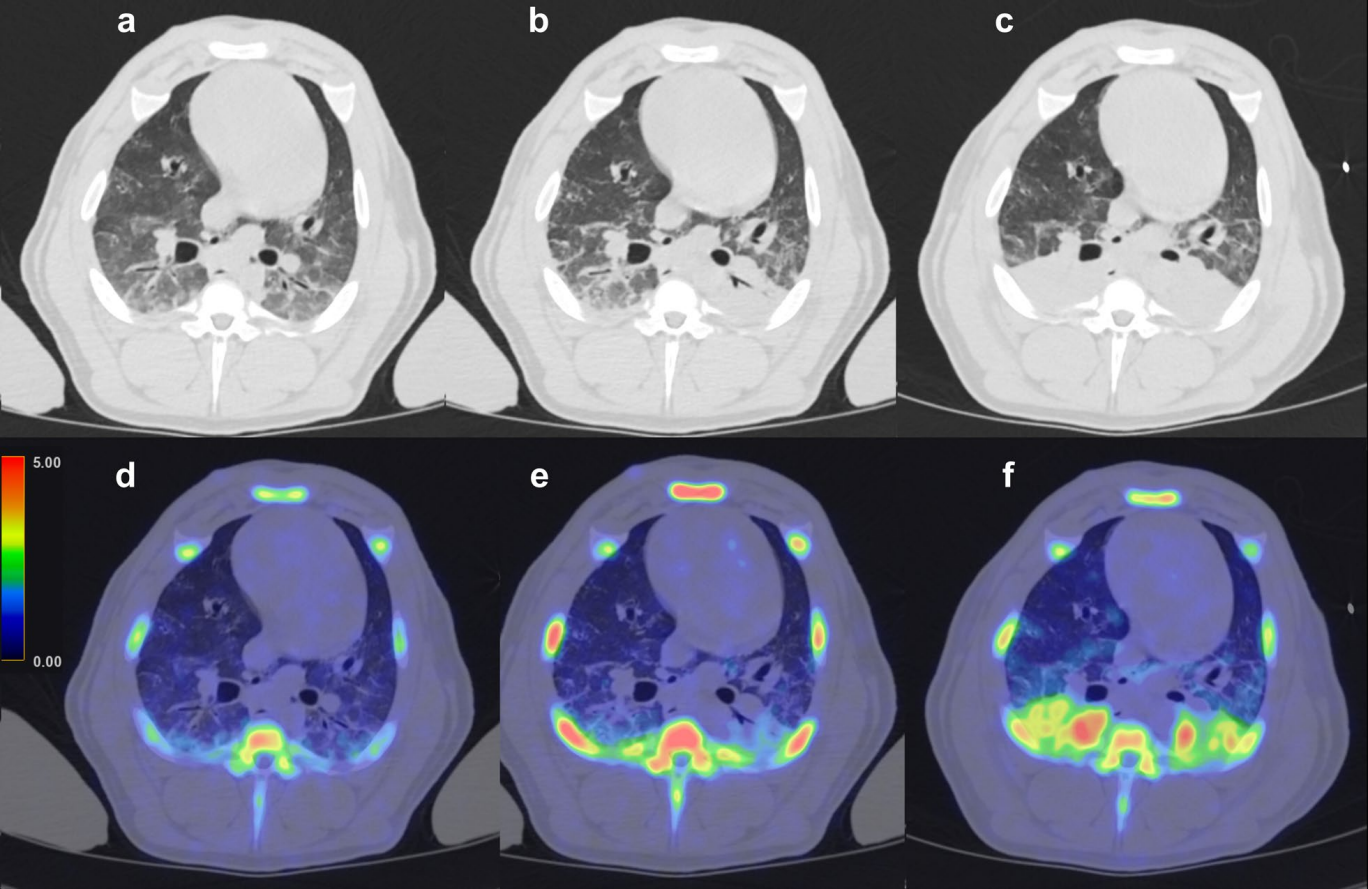
Upper row: trans-axial CT-scans images over the lungs: **a** − 1 h, **b** 2 h, **c** 4 h. Lower row: corresponding trans-axial fused PET–CT images with [^11^C]NES. **d** − 1 h, **e** 2 h, **f** 4 h. All images are corrected to SUV = 5 and thus directly comparable. The amount of NE in the lungs increases as the sepsis progresses

**Fig. 5:**
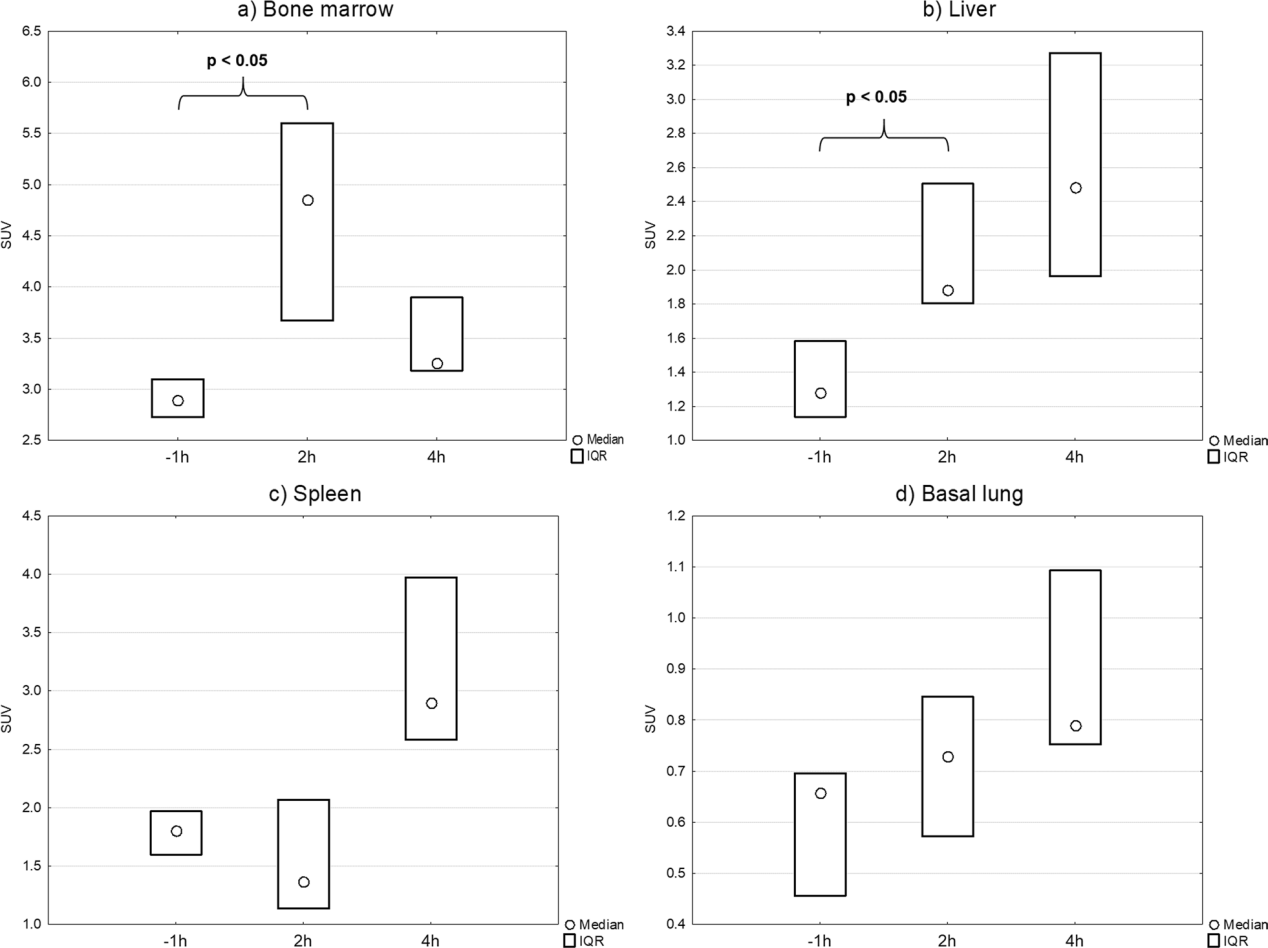
**a**–**d** Median SUV at − 1 h, 2 h and 4 h in: **a** bone marrow,** b** liver, **c** spleen, **d** lung. *n* = 6 at − 1 h and 2 h. *n* = 3 at 4 h. The increase of uptake at 2 h compared to − 1 h in bone marrow and liver is significant using Wilcoxon signed rank test

After sepsis induction the tracer binding in the bone marrow was significantly increased (Figs. 3 and 5A). The uptake of [^11^C]NES in the liver (Figs. 3 and 5B) and lungs (Figs. 4 and 5D) increased over time starting immediately after induction of sepsis while the uptake in the spleen (Figs. 3 and 5C) was clearly increased only later in the sepsis, four hours after sepsis induction. The radioactivity in the entire intestinal region was visibly reduced after sepsis induction, especially at 4 h, likely representing reduced hepatic clearance of labelled metabolites (Fig. 3, representative PET SUV images).

### Total inflammatory volume of the lung

**Fig. 6:**
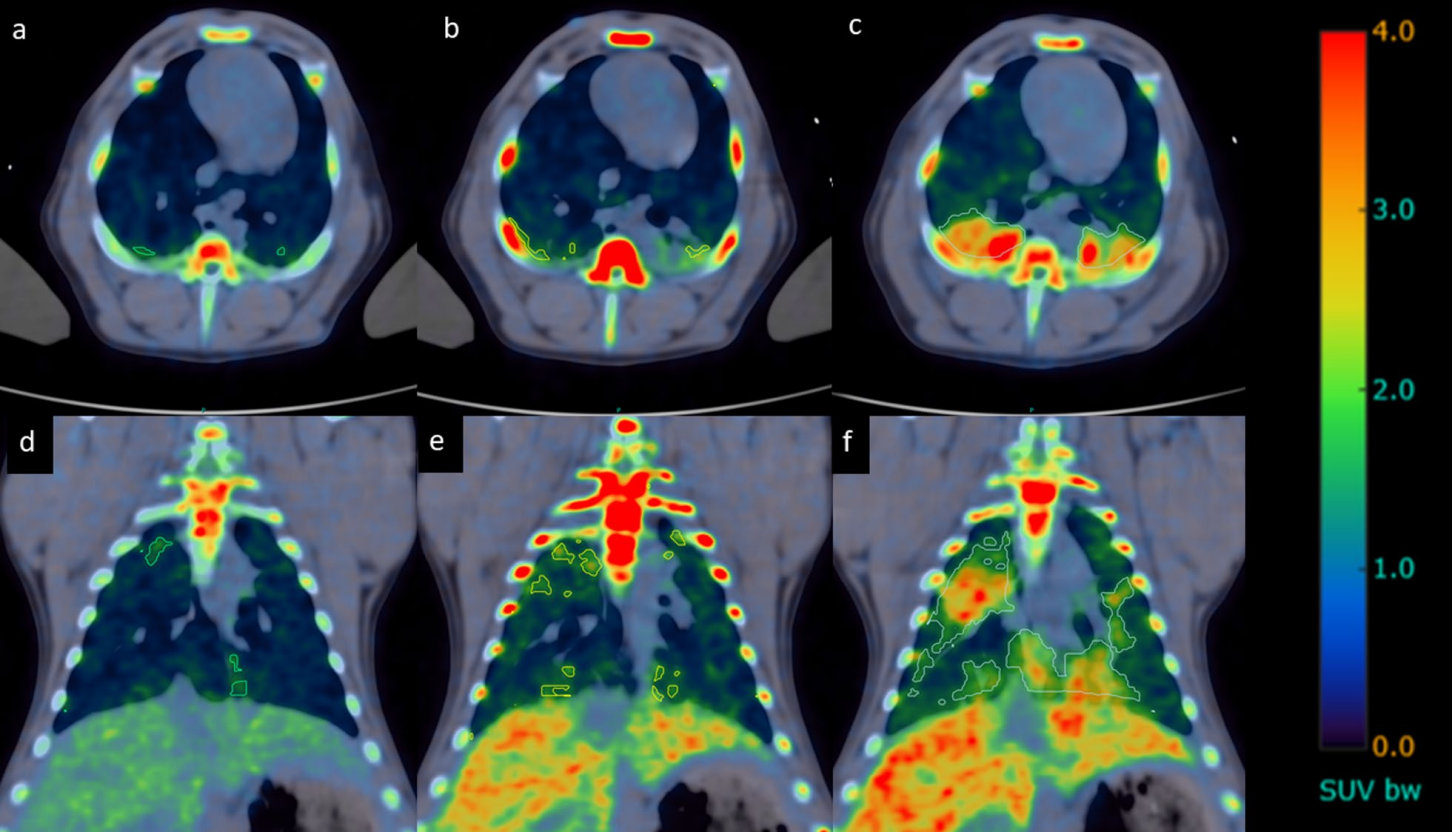
Upper row: trans-axial PET–CT images over the lungs: **a** − 1 h, **b** 2 h, **c** 4 h. Lower row: coronal PET–CT images. **d** − 1 h, **e** 2 h, **f** 4 h. Both the volume of affected lung and the mean SUV of the affected area increase over time, causing the total inflammatory volume of the lungs (TIL) to increase markedly

As seen in Fig. 6, and table S1 (Supplements) both the volume of affected lung and the mean [^11^C]NES SUV increased during the course of sepsis development. The mean TIL at − 2 h, 2 h and 4 h was 7.5 (*n* = 4), 97.4 (*n* = 6), and 349.3 (*n* = 3), respectively. Values for each individual and time are presented in table S1 in Supplements.

### Blocking of NE in vivo

Inhibiting extracellular NE by pre-administration of sivelestat in P6 did not affect the binding in bone marrow, lung spleen or liver, indicating that the binding primarily represents [^11^C]NES binding to NE in intact neutrophils and neutrophil precursors. This animal is thus included in the statistical analysis, since exclusion made no difference.

### Bone marrow microscopy

**Fig. 7:**
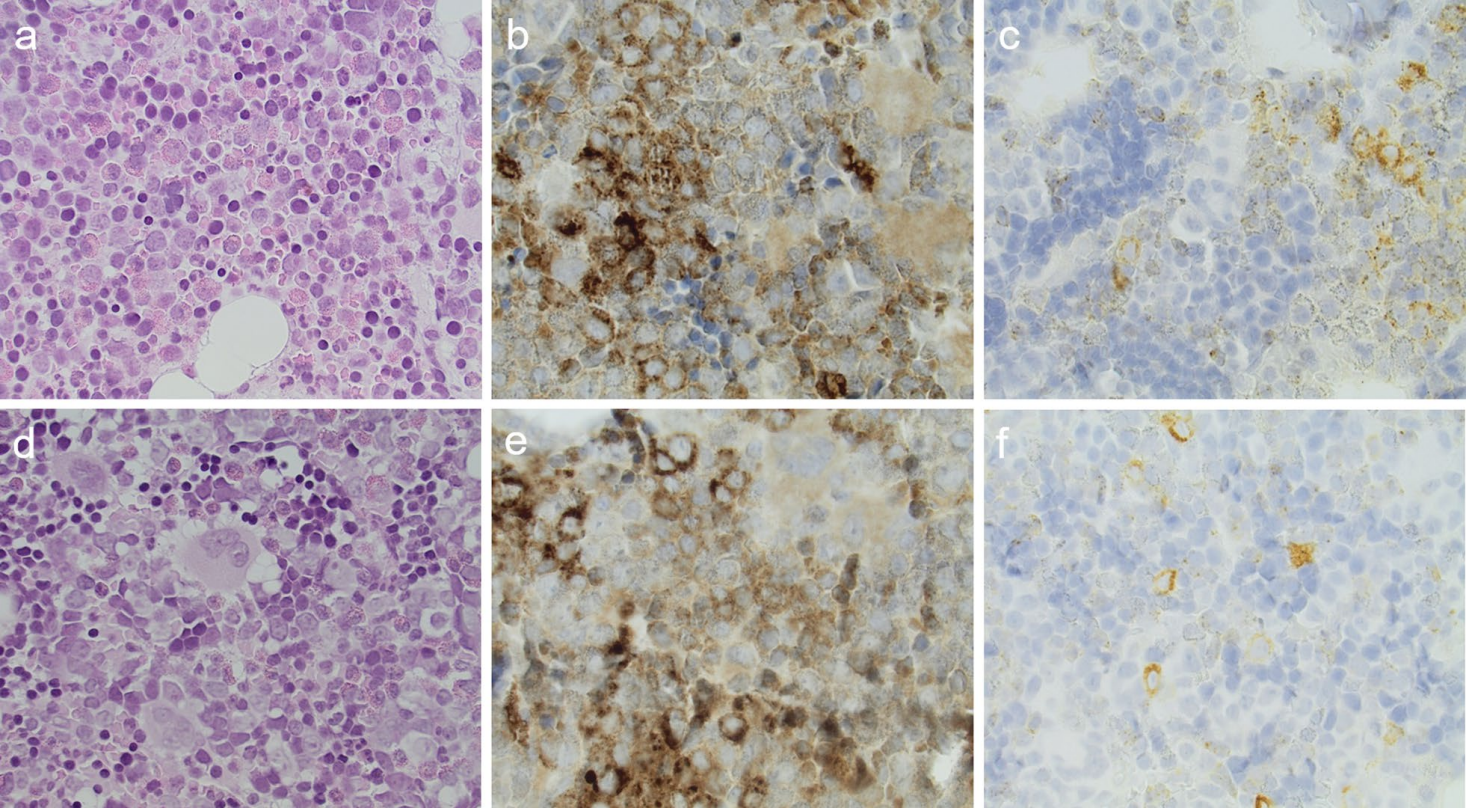
Bone marrow biopsies taken at − 2 h (**a**–**c**) and 3 h (**d**–**f**) from the subject P7 stained with haematoxylin–eosin (a + d), elastase (b + e) and myeloperoxidase (c + f). The cell content and distribution is similar before and after sepsis induction

The femoral bone marrow biopsies from subjects P6 and P7 at − 2 h (before sepsis) and 3 h (after sepsis induction) demonstrated a similar cellular content both regarding bone marrow cellularity and composition of myelopoiesis. The relative amount of different cell types including MPO-positive and NE-positive cells does not differ before and after sepsis (Table 2 and Fig. 7).

**Table 2:** Cell content of the bone marrow and blood

Cells in percent	P6 BM − 2 h	P6 peri − 2 h	P6 BM 3 h	P6 peri 3 h	P7 BM − 2 h	P7 peri − 2 h	P7 BM 3 h	P7 peri 3 h
Blast cells	1		1		2.5		2	
Promyelocytes	1		1		1		1	
Myelocytes	4		7		3		5.5	
Metamyelocytes	13		10		9.5		12	
Band formed cells	23	12	32	40.5	13	16	10.5	32.5
Segmented neutrophils	8	53	2	36.5	6.5	41	1.5	29
Eosinophils	9	5	14	5.5	16	13	25	8
Basophils						1	1.5	2
Lymphocytes	17	25.5	7	16.5	9	23	11	27.5
Monocytes	3	4.5	1	1	1.5	6	< 1	1
Plasma cells					1.5		1	
Sum of erythropoiesis	21		25		36.5		29	
MPO-positive cells	15		8.6		7.1		5.9	
NE-positive cells	34.2		42.6		59.4		32.7	

The relative content of cells (%) in bone marrow (BM) and peripheral blood (Peri) before (− 2 h) and after (3 h) sepsis induction in the two individuals P6 and P7

MPO, myeloperoxidase; NE, neutrophil elastase

### Blocking of NE in purified human neutrophils in vitro

**Fig. 8:**
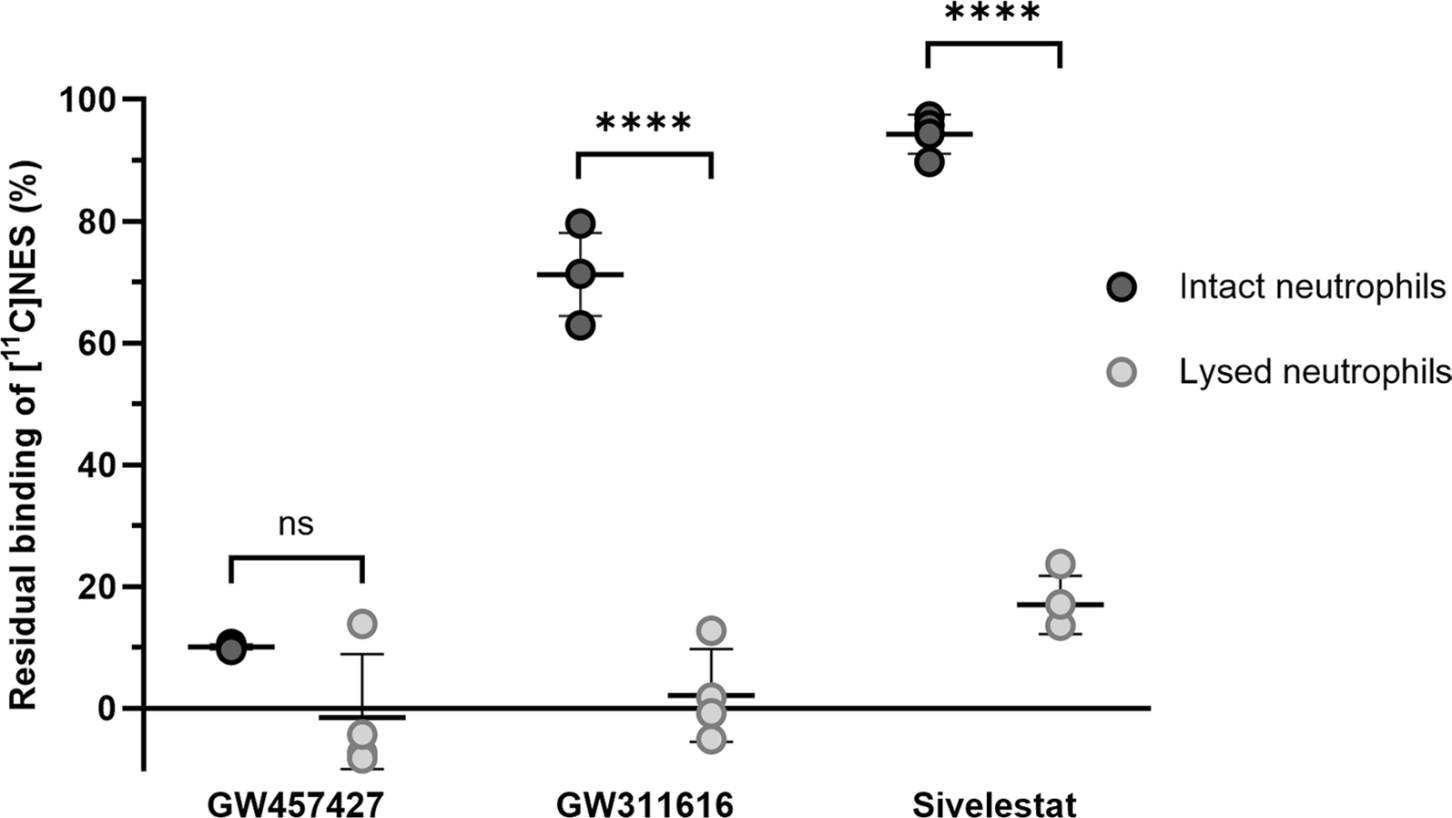
The binding of [^11^C]NES to intact and lysed neutrophils after blocking with the three substances GW457427, GW311616 and sivelestat. Each value is presented in the graph as well as mean ± SD. **** = *p* < 0.0001. Both GW311616 and sivelestat block significantly more in lysed neutrophils than in intact

The residual binding of [^11^C]NES in the presence of an excess of GW457427 was less than 10% of the binding in the absence of blocking compound, both in intact and in lysed neutrophils. Excess of GW311616 was able to achieve 97% blocking in the lysed fraction but only 29% in intact neutrophils, a significant difference, indicating less accessibility to the elastase stored in granule. Sivelestat also demonstrated a significantly lower blocking effect in intact than in lysed neutrophils; 87% blocking in lysed neutrophils compared to only 2% blocking in the intact fraction (Fig. 8).

## Discussion

In this work, we show that the PET-tracer [^11^C]NES can be used in a porcine intensive care sepsis model and visualise the distribution and redistribution of NE cross tissues in the first hours of experimental sepsis. We further imply that the biodistribution of neutrophils can be studied by tracing the biodistribution of NE.

Sepsis is associated with a rapid immunological response initiated momentarily when microorganisms are present in blood. The innate immune response is immediately alerted leading to the convergent and rapid release of chemokines and cytokines that includes TNF-α, leukotrienes, interleukin 1 (IL1), IL6, IL8 and Fas which in turn have a profound impact on neutrophil migration and activation and will also prepare the bone marrow for recruitment of cells to the periphery [23, 24].Since the response of the bone marrow to sepsis is release of an abundance of neutrophils, even immature, the pronounced increase of [^11^C]NES in bone marrow one hour after induction of sepsis is counterintuitive.

The affinity of [^11^C]NES to the precursor NE protein is not known, but it can be expected that the active site is inactive and interaction and binding of [^11^C]NES is markedly lower than for active NE (which [^11^C]NES binds with single digit nanomolar affinity).

The in vitro binding study (Fig. 8) in neutrophils with [^11^C]NES showed that the tracer binds to free NE but also penetrates the cell membrane and bind to NE in intact azurophilic granules as also discussed in previous studies [17]. Sivelestat is known to not penetrate the cell membrane and thus only block extracellularly released NE [25] which is consistent with our in vitro binding result (Fig. 8). Thus, the lack of blocking effect on [^11^C]NES binding in bone marrow, spleen and liver demonstrates that the binding in these tissues is to intracellular NE in intact neutrophils or neutrophil precursors. Since the timeline in our model is only a few hours, it is logical that no substantial amount of granulae degranulation occurred.

The involvement of NETs in sepsis has been recognised as an important defence mechanism against the invading microorganisms, but at the same time also partly responsible for the development of the uncontrolled and devastating ARDS-like inflammatory response in the lungs. NETosis is expected to occur early in sepsis, already at day one, and it cannot be ruled out that NETs were formed in our study, although the lack of blocking effect of sivelestat indicate very low levels of extracellular elastase in most tissues [26].

In the bone marrow biopsies there was no clear demonstration of increased amount of neutrophils, their precursors or the amount of MPO or NE-positive cells due to induction of sepsis. Biopsies were only from two animals, but they support the theory that the increase in [^11^C]NES seen in the bone marrow early in sepsis is not due to the increase in the amount of one specific kind of cell nor due to an increase of NE-positive cells.

We suggest that the early increase in the uptake of [^11^C]NES in the bone marrow is reflecting the rapid crosstalk of released mediators impacting the bone marrow, resulting in the increased signal from intracellular NE in the azurophilic granule. We find it likely that this is a picture of the general activation including maturation of cells and cleaving of NE-precursor into NE. NETs production in the bone marrow seems immunologically inadequate and in this model any extracellular NE component would also be blocked by sivelestat.

The next phase of the early neutrophil-mediated inflammatory response: migration of neutrophils from bone marrow to blood, is seen both in the PET data (Figs. 3, 4h) and in the blood neutrophil count (Fig. 2). This is also associated with increased uptake of [^11^C]NES in liver, spleen and lungs, which indicates migration of neutrophils to organs as an active defence mechanism where pathogens are initially removed by phagocytosis.

The radioactivity in the liver has two potential components, [^11^C]NES uptake in intact neutrophils and radioactive metabolites of the tracer. The significant reduction of radioactive metabolites in the intestines after induction of sepsis could be a result of reduced liver metabolism as part of the ongoing sepsis. Another tentative explanation could be that the most of [^11^C]NES rapidly is bound to increased amounts of activated neutrophils and thereby not metabolised. The very pronounced increase in the spleen also points towards increased amount of [^11^C]NES uptake in circulating neutrophils.

The steady increase of [^11^C]NES uptake in lungs after sepsis is of particular interest. Sepsis is known to cause secondary immune-mediated ARDS, which is one of the major reasons for the high morbidity and mortality [27]. The atelectasis formation in this model typically reach equilibrium within one hour. However, atelectatic lung tissue is usually described as in West´s zone 4, where the blood flow is decreased due to increased interstitial pressure. Thus, the baseline uptake of [^11^C]NES in the dorsal part of the lungs is probably less due to atelectasis than the gravitation-induced ventilation/perfusion (V/Q) mismatch seen in West´s zone 3 in a mechanically ventilated pig in supine position [28]. In Fig. 4, the PET uptake and corresponding CT-images show a gradual increase in [^11^C]NES uptake and a concomitant increase in HU following induction of sepsis. Although a similar pattern would be seen if the blood volume was increased, the acutely decreased cardiac output, in combination with increased MPAP and PVRI leading to a markedly decreased pulmonary blood flow indicate that neither pulmonary blood volume nor blood flow explain the increased [^11^C]NES uptake. Our interpretation is that the [^11^C]NES uptake in the lungs reflect infiltrating neutrophils and ongoing inflammation. This is line with clinical experience in sepsis where lung inflammation is one of the hallmarks [29].

Another interesting clinical correlate worth mentioning is Alpha1-antitrypsin (AAT), the main inhibitor of NE. Patients with AAT deficiency display an increased NE activity. AAT deficiency is a genetic disease primarily affecting the lungs and liver and Alpha-1 patients mainly die due to respiratory failure. This tissue involvement is in agreement with the finding of increased uptake in the liver and lungs in the present study [30]. As described by Sun et al., more in depth research into the mechanistic explanations for the development of sepsis-induced lung injury is highly warranted, with that in mind the current model and methodology could be an important contribution.

The pig as a model of human diseases has a lot of advantages over the mouse, with an immune system resembling ours and a body size that makes intensive care measures possible [31]. We recognise several limitations in our study. The sample size is small, especially for the last PET-scan at 4 h and for the bone marrow biopsies. In a biological system, the hosts’ inflammatory reaction induced by bacteraemia has a large variance, and this notion carries the risk of having a large impact in small group sizes. However, the pigs included in the study are genetically relatively homogenous and the inflammatory inducting dose of bacteria controlled, reducing the variability of the inflammatory reaction. This model induces sepsis by an intravenous infusion of bacteria not comparable to any relevant clinical sepsis. The strength of this model is the knowledge of the exact time point when the reaction starts and the possibility to study the acute phase of the septic reaction. The use of a PET-tracer in a large animal model is a ground-breaking novel way of studying sepsis carrying great potential for future studies.

The understanding of the initial phase of sepsis is incomplete. Our study adds knowledge to the early phase of the innate immune system response in sepsis. We assert that our data show that the early response, with a significantly increased [^11^C]NES uptake in bone marrow in conjunction with a lack of histological evidence of neutrophil infiltration, is a picture of intense neutrophil activation and increased conversion of pro-elastase to elastase in the bone marrow. We further assert that the study has depicted very early sepsis-induced neutrophil accumulation in the lungs in vivo. Both are novel findings that underline the potential of the method to understanding the innate systemic immune reaction better.

## Conclusion

The neutrophil elastase PET-tracer [^11^C]NES can be used to visualise the early neutrophil response in a porcine sepsis model. A pronounced increase of NE was found in the bone marrow during the almost immediate sepsis reaction followed by a gradual increase in the liver, spleen and lungs and a concomitant reduction of the tracer uptake in bone marrow. Dynamic PET-examinations in relevant large animal sepsis models can serve an important part in mechanistic studies of sepsis-induced organ failure, such as in ARDS.

## Supplementary Information

Additional file 1. Table S1. ^11^[C]-NES PET, pigs scanned over the lung, static scan at – 1 h, 2 h and 4 h.

## Data Availability

Data can be sent from the corresponding author upon reasonable request.

## Abbreviations

ARDS: Acute respiratory distress syndrome
Bq: Becquerel
BSA: Body surface area
CFU: Colony forming units
CI: Cardiac index
CO: Cardiac output
CT: Computed tomography
DIC: Disseminated intravascular coagulation
*E. coli*: *Escherichia coli*
FiO_2_: Inspired oxygen fraction in air
GBq: Gigabecquerel
h: Hours
HU: Hounsfield units
ICU: Intensive care unit
IL: Interleukin
i.v.: Intravenous
kg: Kilograms
MAP: Mean arterial pressure
MBq: Megabecquerel
MPAP: Mean pulmonary arterial pressure
MPO: Myeloperoxidase
NE: Neutrophil elastase
NETs: Neutrophil extracellular traps
PaO_2_: Partial pressure of oxygen in arterial blood
PET: Positron emission tomography
PVRI: Pulmonary vascular resistance index
ROS: Reactive oxygen species
SD: Standard deviation
SUV: Standardised uptake value
TIL: Total inflammatory volume of the lungs
TNF-α: Tumour necrosis factor-alfa
