# Supplementary material for: Repeated positron emission tomography tracing neutrophil elastase in a porcine intensive-care sepsis model
Source: Intensive Care Med Exp. 2025 Feb 4;13:14. doi: 10.1186/s40635-025-00721-3 (PMC11794750; doi:10.1186/s40635-025-00721-3)
Supplement: Supplementary file 1 — Additional file 1. Table S1. 11[C]-NES PET, pigs scanned over the lung, static scan at – 1 h, 2 h and 4 h. [file 40635_2025_721_MOESM1_ESM.docx]

Supplements

**Supplement 1 - Intensive care protocol**

At arrival the animals were immediately sedated with 150 mg tilétamine-zolazepam and 55 mg xylocaine. After induction of anaesthesia, intravenous access was established and ketamine 100 mg and morphine 20 mg was given intravenously (i.v.). Anaesthesia was maintained by a continuous i.v. infusion of pentobarbital 8 mg x kg^-1^ x h^-1^ and morphine 0.26 mg x kg^-1^ x h^-1^ solved in 25 mg x mL^-1^ glucose at 8 mL x kg^-1^ x h^-1^. Ringer´s acetate solution was given as an initial bolus of 20mL x kg^-1^ followed by a continuous infusion of 2 mL x kg^-1^ x h^-1^. Rocuronium bromide of 2.5 mg x kg^-1^ x h^-1^ was administered as a continuous i.v. infusion.

The pigs were tracheotomised and mechanically ventilated using a Servo I ventilator with volume-controlled mode and initial settings of inspired oxygen fraction in air (FiO_2_) 30%, respiratory rate 25 min^-1^ and positive end-expiratory pressure (PEEP) 5 cm H_2_O. Tidal volume was adjusted during the upstart period to maintain an arterial carbon dioxide pressure (PaCO_2_) of 5.0-5.5 kPa.

After initiation of anaesthesia and mechanical ventilation a right paratracheal skin incision was made followed by blunt dissection and identification of the internal jugular vein and the thyroglossal artery. Central venous access was then established by catheterisation of the superior caval vein, and a Swan Ganz catheter placed in a pulmonary artery. Arterial access was achieved with a catheter into a branch of the right thyroglossal artery. A small lower laparotomy was performed to reach and catheterize the urinary bladder.

**Supplement 2 – Interventions**

| **Parameter** | **Threshold values for intervention** | **Intervention** |
| --- | --- | --- |
| PaO_2_ | <10 kPa first time | Increase FiO_2_ to 0.6 |
|  | <10 kPa there after | Increase FiO_2_ to the next level 0.6 🡪0.8 🡪1.0 |
|  | >20 kPa | Decrease FiO_2_ to the next level 1.0 🡪0.8 🡪 0.6 🡪 0.45 🡪 0.3 |
| PaCO_2_ | >6.5 kPa | Increase tidal volume by 10% up to 15 mL x kg^-1^ |
|  | <4.5 kPa | If RR ≤25, decrease tidal volume by 10% down to 4 mL x kg^-1^  If RR >25 Decrease RR by 10% |
| MAP | MAP=MPAP at < 90 min after start of bacterial infusion. | Single dose of 40 µg of noradrenaline |
|  | <60 mmHg (50 is used as a threshold at < 90 min after start of bacterial infusion) | Start noradrenaline infusion 0.07 µg x kg^-1^ x min^-1^. If ongoing, increase rate one step: 0.07🡪 0.13🡪 0.29🡪 0.54 µg x kg^-1^ x min^-1^  If CO <2.5 L/min give RA bolus 15 mL x kg^-1^ |
|  | >100 mmHg unless in PET-camera | Decrease noradrenaline to the next step 0.54🡪 0.29🡪 0.13🡪 0.07🡪0 µg x kg^-1^ x min^-1^ |

PaO_2_= arterial partial pressure of oxygen, FiO_2_= inspired fraction of oxygen, PaCO_2_ = arterial pressure of carbon dioxide, RR= respiratory rate, MAP= mean arterial pressure, min= minutes, MPAP= mean pulmonary arterial pressure, RA = Ringer´s acetate solution

**Supplement 3 - Blood sample analysis**

Arterial blood gases were drawn hourly and analysed for oxygen pressure, carbon dioxide pressure, lactate, glucose and haemoglobin in a Radiometer ABL825. Blood or plasma samples were analysed for blood cell count on a Cell Dyn Sapphire cell counter (Abbott Scandinavia) and creatinine (reagent 8L24) on an Architect ^®^ Ci8200 analyser (Abbott Scandinavia). Tumor necrosis factor alpha (TNF-α) and NE were determined by commercial porcine-specific sandwich enzyme-linked immunosorbent assays (DY690B, R&D Systems, Minneapolis, MN, USA and A73750 Porcine Neutrophil Elastase ELISA Kit, antibodies.com, Limited, Cambridge, UK).

**Supplement 4 – Bacterial preparation**

A clinical isolate of *Escherichia coli* (*E. coli*), strain B09-11822 (serotype O-rough:K1:H7) was used. The days before the experiment, the *E. coli* were reinoculated on a cysteine lactose electrolyte deficient plate (CLED) and cultured overnight in 37^o^ C in an incubation chamber. On the morning of the experiment they were grown into log-phase in lysogeny broth medium according to Miller (LB). After a minimum of two hours a portion of the bacteria were removed from the broth and resuspended in saline. The concentration was measured using spectrophotometry and the bacteria was given as an infusion of 8.3 log_10_ colony forming units (CFU) x h^-1^ for three hours. The infusion was changed every hour to assure that the bacteria remained in log-phase.

# **Supplement 5 - In vivo [^11^C]NES PET/CT examinations in pigs**

All PET/CT scans were performed using a Discovery MI system (GE Healthcare) with a 25 cm axial PET field of view (FOV) and a 64-slice CT. The dynamic PET scan was conducted during 60 minutes (34 frames: 1x10, 8x5, 4x10, 2x15, 3x20, 4x30, 5x60, 4x300, 3x600 seconds). Immediately after the dynamic PET scan, a whole-body PET scan was performed over 20-24 minutes (5-6 bed positions depending on the size and positioning of the pig, 4 min/bed position). All PET scans were reconstructed using an iterative VPFX-S algorithm (3 iterations, 16 subsets, 3-mm postfilter, and a 256x256 matrix).

**Supplement 6 - Bone marrow sampling**

The glasses with imprints were manually stained with May Grünwald-Giemsa and the biopsies prepared and stained by the research laboratory at the Swedish National Veterinary Institute in Uppsala and manually counted in the microscope. In addition to standard staining the biopsies were also stained with Dako autostainer Link 48 and Envision FLEX, high pH detection kit Dako (#K8000). Antibodies targeting myeloperoxidase (MPO) Dako (IR511) and ELANE ab68672, rabbit polyclonal; Abcam targeting NE, diluted 1:200 were used.

**Supplement 7 - Binding of [^11^C]NES in purified human neutrophils**

Fresh, whole blood was obtained from healthy donors, which gave oral consent after being informed of the purpose of the experiment. Neutrophils were purified from 2 mL of blood with a commercial kit, based on immunomagnetic negative selection (EasySep™, Human Neutrophil Isolation Kit, STEMCELL Technologies UK Ltd., Cambridge, UK), according to the manufacturer’s protocol. The resulting fraction contained purified neutrophils (~8 x 10^6^cells in ~2.5 mL). From the purified neutrophils, 200 µL were diluted with 800 µL of ice-cold cell extraction buffer (Abcam) and immediately homogenized in a Dounce homogenizer. This sub-fraction was denoted “Lysed”. In parallel, 200 µL of the purified neutrophils were diluted with 800 µL of ice-cold PBS + 1 mM EDTA and denoted “Intact”. The two sub-fractions, containing equal concentration of neutrophils, were incubated in 1 MBq x mL^-1^ [^11^C]NES for 40 min, at room temperature, in the presence or absence of an excess of three elastase inhibitors. The ligands used were NES (10 µmol x L^-1^), GW311616 (2.3 µmol x L^-1^) and Sivelestat (23 µmol x L^-1^). Quadruple samples were used. The incubations were terminated by filtration under vacuum through Whatman GF/C glass fibre filters, using a cell harvester (Brandel, Gaithersburg, MD). The filters were washed three times with 3 mL PBS buffer and transferred to scintillation tubes where the trapped radioactivity was measured in an in-house built scintillation counter of NaI type and corrected for radioactive decay.

**Table S1**

^11^[C]-NES PET, pigs scanned over the lung, static scan at -1h, 2h and 4h.

| SUV | -1h | 2h | 4h |
| --- | --- | --- | --- |
| P 01 | 1.4 | 1.5 | - |
| P 02 | 1.8 | 1.9 | 2.3 |
| P 03 | - | 1.8 | - |
| P 05 | 1.7 | 1.8 | - |
| P 06 | 1.8 | 1.8 | 2.1^†^ |
| P 07 | * | 1.5 | 1.6 |
| SUV * Volume |  |  |  |
| P 01 | 14.6 | 135 | - |
| P 02 | 10.3 | 90.6 | 485 |
| P 03 | - | 103 | - |
| P 05 | 0.8 | 150 | - |
| P 06 | 4.3 | 63.0 | 373^†^ |
| P 07 | * | 42.6 | 190 |
| Volume mL |  |  |  |
| P 01 SUV <1.3 | 10.1 | 88.8 | - |
| P 02 SUV <1.6 | 5.7 | 48.1 | 207 |
| P 03 SUV <1.6 | - | 56.6 | - |
| P 05 SUV <1.6 | 0.46 | 82.3 | - |
| P 06 SUV <1.6 | 2.5 | 34.6 | 177^†^ |
| P 07 SUV <1.3 | * | 28.9 | 120 |
| Hounsfield units ^a^ |  |  |  |
| P 01 | -209 | -281 | - |
| P 02 | -322 | -145 | -111 |
| P 03 | - | -201 | - |
| P 05 | -140 | -75 | - |
| P 06 | -375 | -234 | -438^†^ |
| P 07 | * | -220 | -244 |

^†^ PET scan 4h after sepsis induced pre blocked with sivelestat 400 mg 30 minutes before PET-scan

* No uptake above SUV 1.1 was detected, region not outlined

^a^ Mean of Hounsfield units measured
